# Fibromyalgia diagnosis from a multi-omics approach: a gut feeling

**DOI:** 10.3389/fmicb.2025.1641185

**Published:** 2025-10-02

**Authors:** Elena Durán-González, Jorge Antolín Ramírez-Tejero, Marta Pérez-Sánchez, Carmen Morales-Torres, Rosa Gómez-Morano, Claudia Díaz-López, Antonio Martínez-Lara, David Cotán

**Affiliations:** Pronacera, Sevilla, Spain

**Keywords:** fibromyalgia, diagnostic, proteome, microbiome, mitochondria

## Abstract

**Background:**

Fibromyalgia is a complex disorder whose main symptoms are chronic widespread pain and fatigue and affects between 0.2 and 6.6% of the world population. Nowadays, there are no molecular biomarkers that could facilitate diagnosis. The latest efforts by researchers have focused on studying problems at the level of central nervous system sensitivity, inflammation, and oxidative disorders.

**Methods:**

A total of 892 women were initially enrolled in the study. For individuals who met the inclusion criteria, a plasma proteome analysis was conducted using blood samples. Briefly, blood was collected, centrifuged, and analyzed by liquid nano-chromatography coupled to tandem mass spectrometry. After the raw data analysis, proteins with statistically significant differential abundance and a fold change over 1.2 (20% increase in fibromyalgia compared with control samples) or under 0.8 (20% decrease in fibromyalgia compared with control samples) in fibromyalgia were selected. For fecal metagenome analysis, fecal samples were collected and processed for DNA extraction. Amplicon sequencing of V3–V4 regions from the 16S ribosomal RNA gene was performed using the Illumina MiSeq platform. The statistical analysis was conducted using R v4.3.2 base packages.

**Results:**

After applying exclusion criteria, 242 women (199 patients and 43 age- and environmentally paired controls) provided plasma and feces samples, as well as properly filled health questionnaires. A total of 30 proteins and 19 taxa were differentially expressed in fibromyalgia patients, and their integration into an algorithm allows for discrimination between cases and controls. The multi-omic approach for biomarker discovery in this study proposes a multifactorial connection between gut microbiota and mitochondria-derived oxidative stress and inflammation.

**Conclusions:**

Plasma and fecal multi-omics analysis suggest an intricate and multifactorial connection between gut microbiota and mitochondria-derived oxidative stress and inflammation in FM patients, with glyceraldehyde-3-phosphate dehydrogenase and *Streptococcus salivarius* as leading actors.

**Trial registration:**

NCT05921409.

## Background

Fibromyalgia (FM) is a complex disorder whose main symptoms are chronic widespread pain and fatigue. Frequently, these symptoms are accompanied by a significant variety of related physical and psychological disturbances (Longley, 2006; Clauw, 2014). Despite the lack of knowledge about the etiopathogenesis and the complex diagnosis of the disorder, the prevalence ranges between 0.2 and 6.6% of the world population (Marques et al., 2017), with women accounting for approximately 89% of patients. To date, the non-specificity of the symptoms, the huge increase in worldwide prevalence, and the economic impact that it entails have turned FM into a pressing health and social issue. This evidence, together with the absence of molecular biomarkers that could facilitate diagnosis, underlines the extreme necessity of basic research on this chronic disorder.

Several physical and psychological disturbances such as migraine, muscle cramps, intestinal discomfort, sleep alterations, lack of short- and long-term memory, inability to concentrate, depression, stress, and anxiety, are recurrently reported by FM patients (Valera-Calero et al., 2022). To unveil the source of these symptoms, the latest efforts by the researchers have focused on studying problems at the level of central nervous system (CNS) sensitivity (Giorgi et al., 2023), inflammatory and oxidative disorders (Coskun Benlidayi, 2019), and even imbalances related to the intestinal microbiota (Liu et al., 2023).

Increasing evidence indicates that inflammatory processes occurring in peripheral tissues and the CNS, primarily in the spinal cord and the brain, are responsible for the pathophysiology of FM (Bains et al., 2023; O'Mahony et al., 2021). The release of chemokines and cytokines leads to the activation of both the innate and adaptive immune systems, as well as neuroinflammation (Littlejohn and Guymer, 2018). These mechanisms are reflected in many of the peripheral clinical features reported by FM patients, such as swelling, dysesthesia, cognitive changes, and fatigue. This lack of energy that accompanies FM patients has been related to mitochondrial dysfunction and oxidative stress. In this sense, it has been shown that there is a correlation between oxidative processes and pain sensitization in FM patients. In this way, a deficiency in the function of enzymes such as superoxide dismutase 1 (SOD1), catalase (CAT), and nicotinamide adenine dinucleotide phosphate (NADPH) oxidase has been identified in FM patients (Assavarittirong et al., 2022), which correlates with the severity of pain and fatigue (Rus et al., 2021; Altindag and Celik, 2006). Likewise, certain studies have identified a lower total antioxidant capacity, accompanied by a greater presence of reactive oxygen species (ROS) in women with FM compared to the control group (Rus et al., 2021). As a whole, all these processes cause immunity deregulation and mitochondrial imbalances that, ultimately, can trigger peripheral neuroinflammatory processes (Coskun Benlidayi, 2019).

The gut-brain axis stands out as one of the recent alterations linked to FM. The main hypothesis indicates a connection between intestinal microbiota imbalance and the increase of intestinal permeability in patients, with the consequent alteration in digestion, immune response, and CNS signaling (Erdrich et al., 2020; Goebel et al., 2008). When the intestinal epithelial barrier is compromised, it can lead to increased intestinal permeability, allowing gram-negative bacteria to move through the mucosal lining and reach the blood flow. Their ability to produce neurotransmitters and their interaction with the primary function of the vagus nerve allows these bacteria to modulate the activity of the hypothalamic-pituitary-adrenal (HPA) axis (Tomasello et al., 2018). This situation allows direct interactions between intestinal microbiota components and humoral and cellular mediators from the immune system and the CNS, triggering an immune response characterized by increased production of inflammatory mediators (Moloney et al., 2016). Thus, several studies have observed changes in the composition of the gut microbiota in various disorders, including gastrointestinal, rheumatic, and metabolic conditions (Wang et al., 2022; Afzaal et al., 2022). In the FM context, a recent study has found a reduced bacterial diversity, as well as altered levels of glutamate and serine, suggesting changes in the metabolism of crucial neurotransmitters in the CNS signaling (Clos-Garcia et al., 2019). This innovative approach based on intestinal microbiota opens a new field for searching for biomarkers. Those biomarkers could also be modulated through diet therapy, which has been gaining relevance in FM management.

Advances in identifying inflammatory markers involved in FM have been largely made thanks to the development of omics techniques. Proteomics studies have been conducted in patients, primarily in saliva, serum, and cerebrospinal fluid. Therefore, (Ramírez-Tejero et al. 2018) identified 33 plasma proteins with different expression in FM patients compared with volunteers, with most proteins linked to inflammatory processes. Another study in patients revealed differences in plasma proteins involved in inflammatory, metabolic, and immunological processes and demonstrated a correlation between these proteins, patients' pain perception, and psychological distress (Wåhlén et al., 2020). Additionally, 22 differentially expressed proteins were identified in FM patients compared with pain-free volunteers, mainly associated with blood coagulation processes, immune response, and interactions with extracellular matrix receptors (Han et al., 2020). Among other findings, these results confirmed the promising utility of proteomics in the study of FM and demonstrate the roles of coagulation, inflammation, and the immune response in the disorder's pathogenesis.

In spite of the above-mentioned research, nowadays, the diagnosis and treatment of FM remain controversial. Traditionally, the diagnostic criteria of the American College of Rheumatology (ACR) have been followed (Wolfe et al., 1990, 2011, 2016), and most healthcare professionals treat their patients with numerous drugs focused on symptoms such as pain, depression, and fatigue. However, clinical recommendations from the European League Against Rheumatism (EULAR) and other organizations point to a multidisciplinary point of view of FM and its treatment (Giorgi et al., 2023; Macfarlane et al., 2017). Likewise, the latest trend is toward the use of highly individualized therapies combined with non-pharmacological approaches indeed (Kundakci et al., 2022).

In this context, this study is focused on biomarker discovery for FM in plasma and feces from 199 FM patients and 43 women as a control group, aiming to combine the analysis of both samples in a diagnostic panel for clinical practice.

## Methods

### Cohort recruitment

#### Study population

To calculate the size of the cohort, the most comprehensive data on the estimated prevalence of FM in Spain published in 2008 were considered (Mas et al., 2008). These authors concluded that approximately 2.4% of the Spanish population was suffering from FM. Specifically, this prevalence reached 8.4% in the age range of 40–49 years, whereas in women aged 50–59, it was 6.7%. A sample size calculation was performed according to the protocol described by (Naing et al. 2006), with a 95% confidence interval (CI) and an α level of 0.05. The result of this sample size calculation was n = 198 (188 plus a 5% of the estimated withdrawal), which means that the cohort had to comprise at least 198 patients with FM to obtain adequate statistical power.

Participants were recruited, and all samples were collected between March 2020 and September 2020. Both FM patients and volunteers were Caucasian. Before the beginning of the project, participants completed an informed consent form and a patients' information datasheet that have been approved by the Ethical Committee for Research with Medications (CEIM) at the Quirónsalud-Catalunya Hospital Group (protocol code IDI-20210749, approval record No. 01/2022).

#### Inclusion criteria

Individuals included in the study were selected through an online survey sent to FM associations, allowing for effective recruitment and reaching a larger pool of potential participants. All the personal and clinical data were recorded and processed as required by the Spanish law (Organic Law 3/2018, of December 5th) (Jefatura del Estado, 2018). A total of 892 questionnaires were received with clinical information from potential patients and control subjects, who were subsequently selected according to the following inclusion criteria for both groups:

- Women within an age range of 40–59 years, since these women are the most affected population group in Spain (Mas et al., 2008).
- Individuals with a BMI >18.5 and < 35.9.

As for exclusion criteria for both groups, the following conditions were established:

- Not being in the age or BMI range.
- Suffering from any type of cancer.
- Individuals who have undergone antibiotic treatment during the 30 days before the first sample extraction.

Additionally, for FM participants, a confirmed FM diagnosis by a healthcare professional, as defined by any ACR criteria, was required.

Although they were not considered exclusion criteria, clinical data on comorbidities and lifestyle habits were collected for analysis as potential confounding factors. Additional information was gathered about cardiovascular diseases (e.g., atherosclerosis, cardiomyopathy), autoimmune diseases (e.g., lupus, celiac disease, thyroiditis), or metabolic disorders (e.g., diabetes, metabolic syndrome). Besides, questions about pharmacological treatment with non-steroidal anti-inflammatory drugs, analgesics, antidepressants, antioxidants, lifestyle habits, diet, smoking, and alcohol consumption were also included in the questionnaires.

### Clinical data collection

All clinical and personal data collected in this study were handled confidentially and in compliance with applicable data protection regulations. Technical and organizational measures were implemented to ensure the anonymization of samples and the dissociation of any identifying information. Access to clinical information was restricted to authorized personnel and stored on secure servers with access control.

The participants answered two clinical questionnaires related to their health status. All of them have completed the Spanish version of the 36-item Short Form Survey health questionnaire (SF-36) following the methodology described by (Vilagut et al. 2005). This questionnaire assesses the physical and psychological wellbeing of the participants. The 36 questions cover eight specific scales: physical functioning (PF), role limitations due to physical health (PH), pain (P), general health perceptions (GH), vitality (V), social functioning (SF), role limitations due to emotional problems (EP), and mental health (MH). The results were interpreted considering a higher score to be a better state of health.

Furthermore, only FM patients completed the revised FM Impact Questionnaire (FIQR) to assess the impact of FM on their daily lives. This questionnaire evaluates physical functioning, work performance (missed workdays and work difficulty), depression, anxiety, morning fatigue, pain, stiffness, fatigue, and overall wellbeing during the preceding week. Its 21 items are divided into three main domains: function (*n* = 9), overall impact (*n* = 2), and symptoms (*n* = 10). Each item is scored on a scale of zero (no impairment) to 10 (maximum impairment), and the total function score is divided by three. The sum of the scores for symptoms is divided by 2, and the overall impact score remains unchanged. The total score of the FIQR has been calculated by adding these three scores, with the maximum possible total score being 100. All the participants' recruitment and clinical data collection are detailed in our clinical data registration (NCT05921409) (Pronacera. Clinicaltrials, 2024) and protocol study already published by (Lucena Del Amo et al. 2023).

### Antioxidant capacity measurement

The total antioxidant capacity of participants was determined by electrochemistry, as described in (Lucena Del Amo et al. 2023).

### Plasma proteome analysis

Whole blood samples were collected in heparin-lithium tubes through venipuncture performed by a healthcare professional. To isolate the plasma fraction, the sample was transferred to a 15 ml Falcon tube and then centrifuged at 2,300 RCF for 10 min at 4 °C. Then, plasma was aliquoted and stored at −80 °C until batch analysis. Before mass spectrometry analysis, high-abundance proteins were depleted using Thermo Fisher Top 14 columns, following the manufacturer's instructions. Depleted samples were digested with trypsin and desalted before injection. To analyze the proteome, we performed liquid nano-chromatography coupled to tandem mass spectrometry (nLC-MS/MS) using an EVOSEP ONE system, which was coupled to the TIMS Tof Pro hybrid mass spectrometer. This analysis was carried out through the services provided by the BioGUNE Cooperative Research Center in Biosciences, located in the Basque Country, Spain. In brief, 250 ng of the sample were injected into the system. The 60 samples-per-day (SPD) protocol was used, with a 22-min chromatographic gradient from 5% to 50% acetonitrile [B], followed by a 2-min wash phase at 85% [B]. Acquisitions were performed on a TIMS TOF Pro system using the standard short-gradient method provided by Bruker in data-dependent acquisition (DDA) mode.

The processed data were analyzed with the search engines MASCOT (MatrixScience) and PEAKS (Bioinformatics Solutions Inc). Label-free quantification of differential proteomics was also performed by PEAKS (Bioinformatics Solutions Inc.) and MaxQuant (Cox Lab) software. The parameters were optimized considering: tolerances of 20 ppm and 0.05 Da for peptides and fragments, respectively; carbamidomethylation of cysteine considered as a fixed modification, and oxidation of methionines as a variable; Uniprot/Swissprot database limited to *Homo sapiens* as the organism for the searches; and selection of proteins identified with at least one unique peptide with False Discovery Rate (FDR) < 1%. Finally, raw abundances were normalized by Cyclic Loess using the NormalizerDE R package and imputed using the K-nearest neighbor algorithm matrix-wise. A differential abundance analysis was performed using the R package limma. Proteins with statistically significant differential abundance and a fold change (FC) over 1.2 (20% increase in FM compared with control samples) or under 0.8 (20% decrease in FM compared with control samples) in FM were analyzed using the Ingenuity Pathways Analysis software (IPA; Ingenuity^®^ Systems, http://www.ingenuity.com). This bioinformatics application enables the functional analysis of differential expression data from different omics, integrating these data into molecular pathways to predict their activation state and link associated regulatory proteins.

### Fecal metagenome analysis

Fecal samples were collected by participants using the DANASTOOL sample collection kit (Danagen; Barcelona, Spain) and the Fe-Col^®^ Faecal sample collection paper (Alpha Laboratories; Hampshire, United Kingdom) as a stool-catch tool. The provided stool tube contains a DNA/RNA stabilization solution that allows samples to be stored at room temperature until batch analysis. For DNA extraction from fecal samples, the DANAGENE MICROBIOME FECAL DNA KIT (Danagen; Barcelona, Spain) was used. Briefly, 1 ml of the homogenized sample was transferred to a bead tube to ease sample disruption. Then, samples were incubated at 70 °C for 10 min. After the incubation step, thorough vortexing and centrifugation steps were applied. The upper phase was then transferred to a filtering column to extract DNA by centrifugation. DNA quality was assessed with a ThermoFisher Multiskan SkyHigh UV/Vis microplate spectrophotometer (Thermo Fisher Scientific, USA). Optimal concentrations were considered to be greater than 5 μg/μl, and 260/280 and 260/230 ratios were considered to be between 1.8 and 2.1. The resultant DNA extracts were stored at −20 °C until further analysis.

Amplicon sequencing was performed using the Illumina MiSeq platform at HelixBioS facilities (Madrid, Spain), where amplicons of V3–V4 regions from the 16S ribosomal RNA gene (16S rRNA) were sequenced. According to the manufacturer's instructions (Illumina, n.d.), tagging and barcoding processes suitable for Illumina technology were conducted. Pretreatment of the samples was performed using quantitative real-time PCR, followed by further individual quantification, pooling, washing, and titration steps. The sequencing was performed on fragments with sizes ranging from 2 × 250 to 2 × 300, in paired-end format, generating between 150,000 and 200,000 reads per sample. Quality control procedures were implemented using MultiQC software (Ewels et al., 2016), with filtering thresholds set at 50,000 reads per sample, Q30 Phred Score, and an average trimmed read length of 280 bp. After data pre-processing, sequencing results were analyzed with USEARCH V11.1. Briefly, the reads were cleaned up using the UCHIME algorithm to eliminate “singletons” (reads with only one sequence), chimeric sequences, and possible artifacts. Zero-radius Operational Taxonomic Unit (zOTU) clustering was performed using the UNOISE algorithm at a 99% identity (similarity). The sequences were then aligned against the taxonomic database specifically developed for the study of the intestinal microbiota by HelixBioS, HUMAN GUT 16S v3.2024 (HelixBioS), with an alignment cut-off point at 99% (identity) using the algorithm USE-LOCAL. The resulting taxonomic count tables were processed using the R packages DESeq2 (Love et al., 2014) (for differential abundance) and Vegan (Oksanen, 2025) (for diversity analysis). The microbiome functional profile was inferred using PICRUSt (v. 2.4.1), a bioinformatics software package designed to predict the functional content of a metagenome from identified bacteria. The zOTU table, filtered for less than 15% missingness and converted to *biom* format using Qiime2, underwent stratified analysis to determine zOTU contributions. Differential abundance was assessed using ggpicrust2 with DESeq2, and plotting functions were customized. Contribution data were processed with Tidyverse (R base package) to calculate the mean and standard error.

### Statistical and bioinformatic analysis

The statistical analysis was conducted using R v4.3.2 base packages. Data were expressed as the sample mean ± standard deviation (SD). The null hypothesis (H_0_) was defined as no difference between the compared groups, with statistical significance set at 0.05 (α = 0.05). The Kolmogorov-Smirnov test was performed to determine the data distribution before each comparative analysis, thus checking whether the data followed a normal distribution. Depending on the result, either Student's *t*-test for unpaired samples or the non-parametric Mann–Whitney *U*-test was used to compare the difference between the means of groups. Pearson's chi-squared and Fisher's exact test were used for qualitative variables.

Spearman's correlation coefficients (*rho*, ρ) were calculated on FM and control samples separately using normalized protein and taxonomic relative abundances and clinical data. To assess differences in correlation between FM and control samples, Fisher z-tests were conducted, and *p-*values of less than 0.05 were considered statistically significant. Correction for multiple comparisons was implemented through Benjamini-Hochberg FDR.

To evaluate the diagnostic performance of selected omics features, we implemented a deep neural network using the keras package in R. Three datasets were analyzed: Statistically significant proteomic markers (*p* < 0.05), statistically significant metagenomic markers (*p* < 0.05) and a combined dataset integrating both. Features were scaled to the [0, 1] range, and microbial abundances were further binarized using a 0.01 threshold. The model applied was a fully connected multilayer perceptron (MLP) with four hidden layers, trained using the Adam optimizer (learning rate = 0.01) and binary cross-entropy loss. To control class imbalance, sample weights were calculated dynamically from each fold. Training was conducted over a maximum of 800 epochs, with early stopping (patience = 250, min_delta = 0.01) based on validation AUC. Model validation was performed using 10 repetitions of five-fold stratified cross-validation, ensuring balanced class distribution across folds.

AUC was used as the primary performance metric, calculated with the pROC package. Individual ROC curves were generated for each fold and repetition using the *roc* function. To compute the mean ROC curve, the sensitivity and specificity values across all 50 runs (five folds × 10 repeats) were interpolated. The final ROC plots include the mean curve ± 1 standard deviation. This methodology was applied to the three datasets independently (proteomics only, metagenomics only, and combined).

## Results

### Participants characteristics

A total of 892 potential participants were screened for this study. Of these, 642 participants were excluded during the screening phase following the inclusion/exclusion criteria. Seven participants who did not complete all the clinical questionnaires and one who did not deliver fecal samples were also excluded. The final cohort consisted of 242 women, including 199 who were FM patients and 43 who were age- and environmentally paired controls who did not present any symptom related to FM. The characteristics of the study cohort are shown in Table 1. There are no significant differences between groups for any clinical variable (*p* < 0.05), including BMI, physical activity, or tobacco consumption. Nevertheless, regarding comorbidities, a higher but not statistically significant incidence of cardiovascular and metabolic diseases, as well as higher consumption of antioxidants, was found in patients compared to the control group.

**Table 1:** Cohort demographic characteristics.

**Variable**	**Controls (*N* = 43)**	**Patients (*N* = 199)**	***p*- value**
Age (mean)	49.46 ± 8.68	52.19 ± 7.09	0.08
BMI (mean)	25.44 ± 3.96	26.23 ± 4.22	0.19
Smoker (%)	11.63%	16.58%	0.56
Sedentarism (%)	46.51%	54.27%	0.45
Autoimmune disease (%)	6.98%	14.07%	0.31
Cardiovascular disease (%)	4.65%	16.58%	0.08
Metabolic disease (%)	4.65%	14.07%	0.15
Antioxidant consumption (%)	0%	8.04%	0.08
Antibiotic consumption (%)	0%	0.50%	1

Depending on the variable of study Mann–Whitney *U*, Pearson's chi-squared, and Fisher's exact test were used, considering *p-*value < 0.05 as the threshold to establish a significant difference.

Data from questionnaire scores for each study group are shown in Table 2. FM impact was evaluated by FIQR, where patients obtained a 65.94 ± 17.78 score. Both groups (FM vs. C) completed the SF36 questionnaire, in which all the resulting scores on the eight items evaluated showed statistically significant differences (*p*-value < 0.0001) between groups. Particularly, the biggest differences were found in body pain and physical health of patients, whose scores are 77.21 ± 23.02 vs. 20.94 ± 17.77 and 82.56 ± 31.19 vs. 10.88 ± 24.69, respectively.

**Table 2:** Questionnaire scores.

**Variable**	**Controls, C (*N* = 43)**	**Patients, FM (*N* = 199)**	***p*-value**
FIQR score	–	65.94 ± 17.78	–
SF36 physical functioning (PF)	88.02 ± 17.19	44.20 ± 22.91	< 0.0001
SF36 physical health (PH)	82.56 ± 31.19	10.88 ± 24.69	< 0.0001
SF36 emotional problems (EP)	82.95 ± 33.23	38.00 ± 45.71	< 0.0001
SF36 mental health (MH)	59.19 ± 23.18	18.60 ± 14.62	< 0.0001
SF36 vitality—energy/fatigue (V)	71.72 ± 17.31	47.72 ± 19.85	< 0.0001
SF36 social function (SF)	85.17 ± 20.75	37.88 ± 25.89	< 0.0001
SF36 pain (P)	77.21 ± 23.02	20.94 ± 17.77	< 0.0001
SF36 general health (GH)	67.56 ± 18.28	46.23 ± 13.50	< 0.0001

Depending on the variable of study Mann–Whitney U, Pearson's chi-squared, and Fisher's exact test were used, considering p-value < 0.05 as the threshold to establish a significant difference.

### Antioxidant capacity

The total antioxidant capacity of participants showed no statistically significant difference between groups (Supplementary Table S1).

### Proteomic profile of plasma

#### Differentially expressed proteins

The proteomic profile of plasma samples from controls and patients with FM show more than 600 proteins identified. Of these, a total of 242 proteins were clearly detected in both groups. Using a cutoff *p-*value < 0.05 for statistical significance, a subgroup of 30 proteins were found to be differentially expressed. Finally, applying a cutoff value of 20% of FC, 14 were overexpressed (FM/C ratio ≥ 1.20) and 7 were underexpressed (FM/C ratio ≤ 0.83; Table 3). Technical validation of some of these proteins was performed by ELISA determination (Supplementary Table S1) as described in manufacturers' instructions.

**Table 3:** Proteins are differentially expressed in the plasma of patients with FM.

**UNIPROT ID**	**Protein name**	**Abbreviation**	**Ratio (FM/C)**	***p*-value**
P25311	Zinc-alpha-2-glycoprotein	ZA2G	2.05	0.013
P09172	Dopamine beta-hydroxylase	DOPO	1.78	0.019
P60709	Actin cytoplasmic 1	ACTB	1.59	0.003
Q92954	Proteoglycan 4	PRG4	1.58	0.016
Q9C075	Keratin type I cytoskeletal 23	K1C23	1.45	0.034
P05109	Protein S100-A8	S10A8	1.43	0.044
P05106	Integrin beta-3	ITB3	1.42	0.011
P07476	Involucrin	INVO	1.39	0.020
O14732	Inositol monophosphatase 2	IMPA2	1.38	0.006
O14818	Proteasome subunit alpha type-7	PSA7	1.37	0.005
O75342	Arachidonate 12-lipoxygenase 12R-type	LX12B	1.33	0.037
P02749	Beta-2-glycoprotein 1	APOH	1.32	0.013
P06727	Apolipoprotein A-IV	APOA4	1.25	0.003
P31944	Caspase-14	CASPE	1.24	0.022
P08603	Complement factor H	CFAH	1.15	0.017
P02760	Protein AMBP	AMBP	1.10	0.011
P07358	Complement component C8 beta chain	CO8B	0.89	0.013
P03952	Plasma kallikrein	KLKB1	0.88	0.014
P0C0L5	Complement C4-B	CO4B	0.86	0.026
P43251	Biotinidase	BTD	0.86	0.047
P22352	Glutathione peroxidase 3	GPX3	0.85	0.010
P06276	Cholinesterase	CHLE	0.85	0.023
P08519	Apolipoprotein(a)	APOA	0.83	0.026
P04406	Glyceraldehyde-3-phosphate dehydrogenase	G3P	0.82	0.028
P07360	Complement component C8 gamma chain	CO8G	0.81	0.000
P20742	Pregnancy zone protein	PZP	0.78	0.048
P01834	Immunoglobulin kappa constant	IGKC	0.78	0.049
P00742	Coagulation factor X	FA10	0.72	0.005
Q02413	Desmoglein-1	DSG1	0.60	0.012
Q9HDC9	Adipocyte plasma membrane-associated protein	APMAP	0.57	0.032

Mann–Whitney test was used, considering p-value < 0.05 as the threshold to establish a statistically significant difference.

#### Functional analysis of plasma proteome

**Figure 1:**
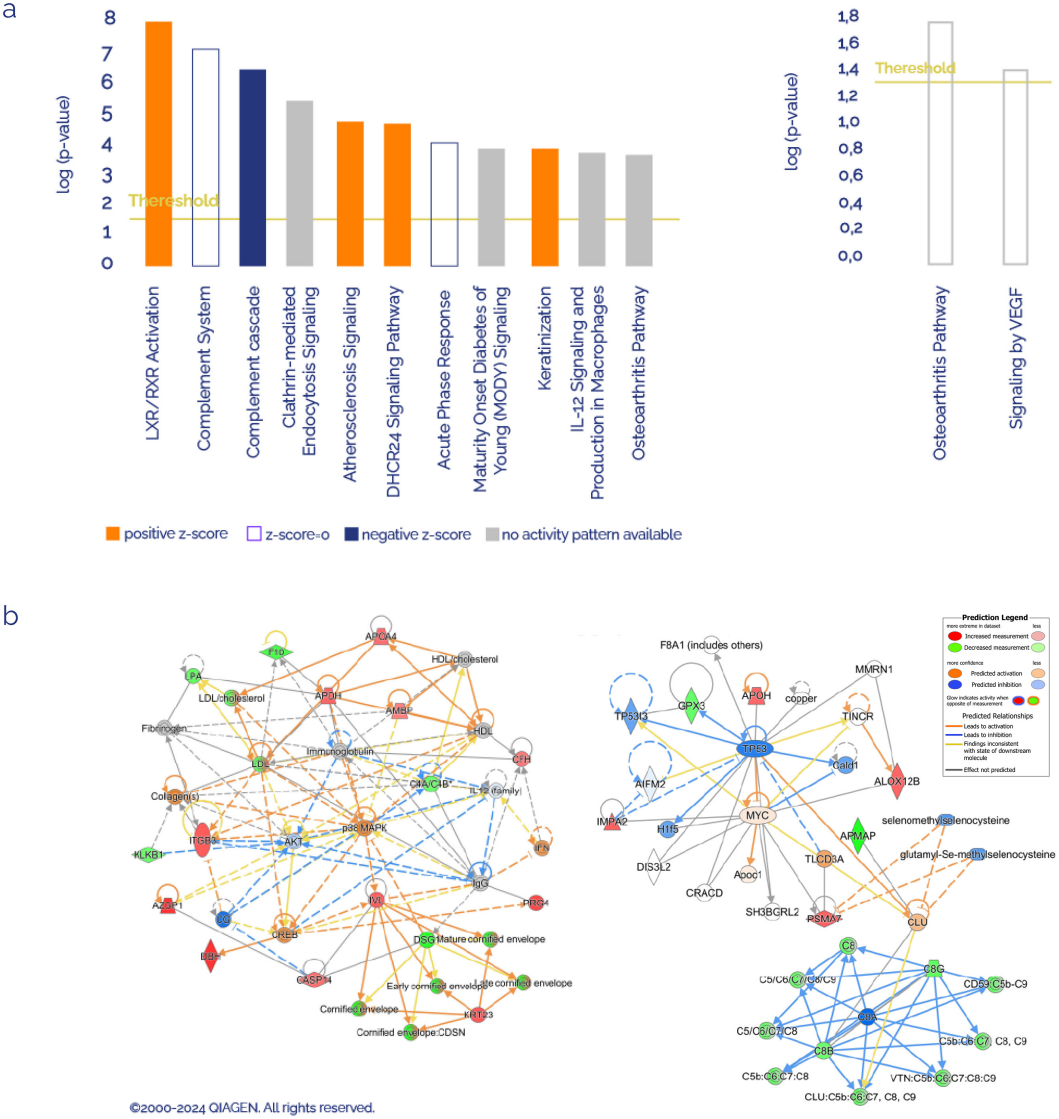
Ingenuity Pathways Analysis of differentially expressed proteins. **(a)** Canonical pathways enriched in FM sorted by significance value, using the Ingenuity Knowledge Database as reference on the left and using the identified list of proteins as reference set on the right. Orange or blue colors mean positive or negative *Z*-score, respectively. **(b)** Most significant molecular networks identified (red FC > 1.2, green FC < 0,8).

IPA software was used to determine whether the proteins with different abundances were related to specific molecular pathways and networks. We analyzed the 30 deregulated plasma proteins found in FM patients, considering as reference set first, the Ingenuity Knowledge Database, and second, all the datasets obtained from plasma samples of this study, to get rid of sample-type bias. Figure 1a shows the most statistically significant enriched canonical pathways. Among these, the top 5 according to the *p-*value with Ingenuity Knowledge Data Base as reference set were Liver-X Receptor/Retinoid-X Receptor (LXR/RXR) activation (six proteins: AMBP, APOA4, APOH, C4A/C4B, LPA and S100A8), Complement System (four proteins: C4A/C4B, C8B, C8G, and CFH), Complement Cascade (five proteins: C4A/C4B, C8B, C8G, CFH, and IGKC), Clathrin-mediated Endocytosis Signaling (five proteins: ACTB, APOA4, ITGB3, LPA, and S100A8), and Atherosclerosis Signaling (four proteins: ALOX12B, APOA4, LPA, and S100A8). Using the study-identified proteins as a reference set, the top canonical pathways were Osteoarthritis Pathway (four proteins: CASP14, ITGB3, PRG4, and S100A8) and signaling by VEGF (2 proteins: ACTB and ITGB3).

In Figure 1b, the top networks with the highest number of proteins involved were shown. On the left network, proteins associated with the inflammatory response, hematological system, and cell-to-cell signaling were gathered using the Ingenuity Knowledge Database as a reference set, where most of the proteins belong to the complement system. On the right network, using all the study-identified proteins as a reference set, most proteins and processes are associated with cancer, organismal development, and reproductive system development and function.

### Metagenomic profile of feces

#### Differentially expressed taxa

A total of 6,030 zOTUs were identified through metagenomic profile analysis of feces samples, capturing 21,639,488 reads. All identified zOTUs were assigned to 696 different species, which were further grouped into 224 genera, 82 families, and 11 phyla.

**Figure 2:**
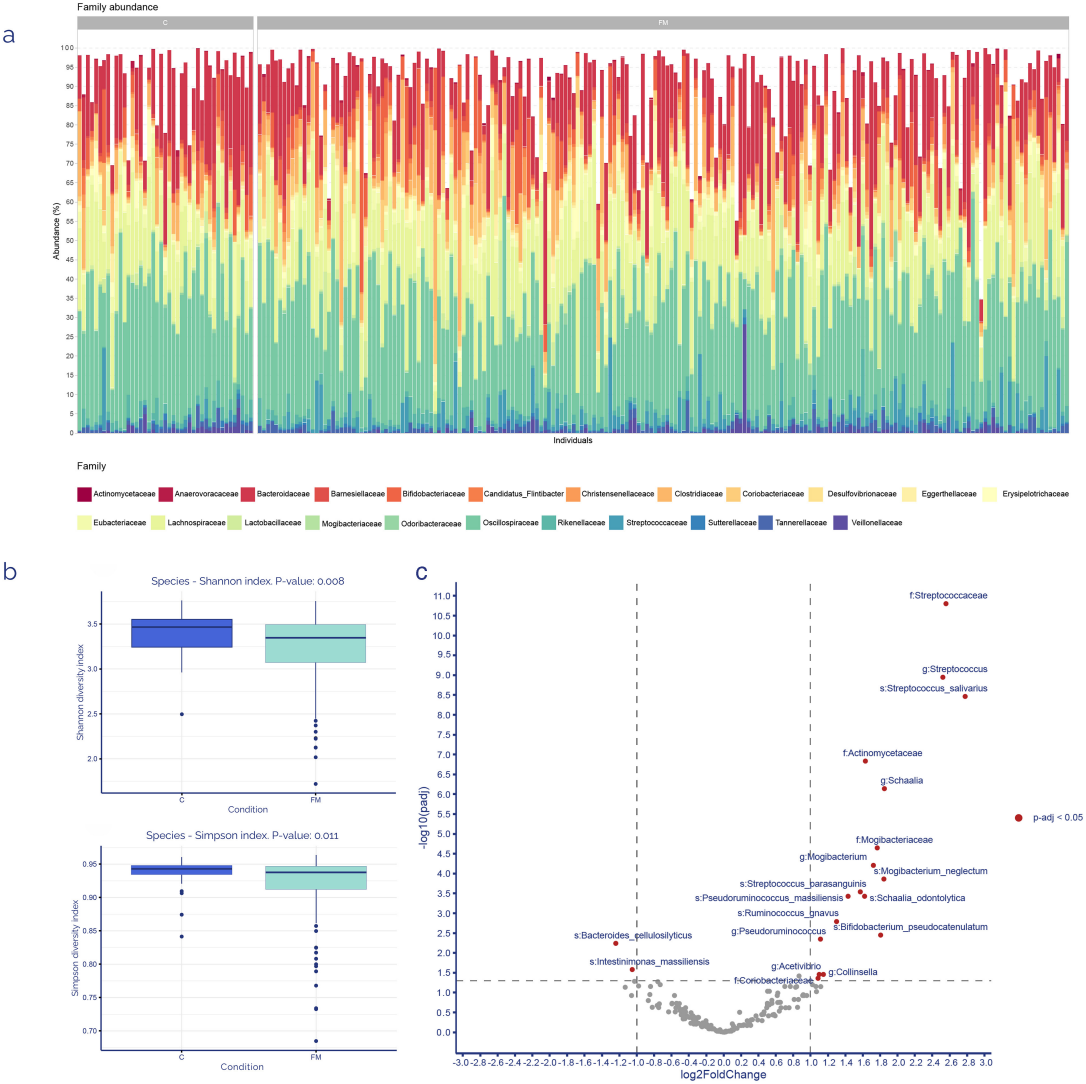
Core microbiome and discriminant analyses. **(a)** Family-level relative taxonomic composition of the gut microbiota among FM patients (*n* = 199) and controls (*n* = 43). **(b)** α-diversity indexes for each sample group at the species-level taxonomic classification, showing the *p-*value computed using the Mann–Whitney *U*-test. **(c)** Volcano plot of 194 taxonomic relatives' abundances detected in feces samples after ubiquity and representativity filtering. Positive log_2_FC indicates increased abundance, and negative log_2_FC indicates a reduction in FM patients. All *p-*values were adjusted using the Benjamini-Hochberg method.

After ubiquity and representativity filtering, the top five most predominant zOTUs families across all samples from both groups were Oscillospiraceae (28% of total raw counts), Lachnospiraceae (17%), Bacteroidaceae (14%), Clostridiaceae (6%), and Bifidobacteriaceae (5%), as indicated in Figure 2a.

Diversity was analyzed throughout 2 α-diversity scores (Shannon and Simpson), with a statistically significant decrease for both scores in FM patients (Figure 2b). Additionally, a differential zOTUs analysis (employing DESeq2) between the controls and FM core microbiomes was performed. The results showed 19 zOTUs whose abundance differed between both groups with an adjusted *p-*value of 0.05 (Figure 2c). Those zOTUs with higher FC in FM patients belonged mainly to Streptococcaceae, Actinomycetaceae and Mogibacteriaceae families. On the contrary, those with negative FC were just two species: *Bacteroides cellulosilyticus* and *Intestinimonas massiliensis*. Technical validation of some of these bacteria was performed by qPCR determination (Supplementary Table S1) as described in manufacturers' instructions.

#### Functional analysis of intestinal microbiota

**Figure 3:**
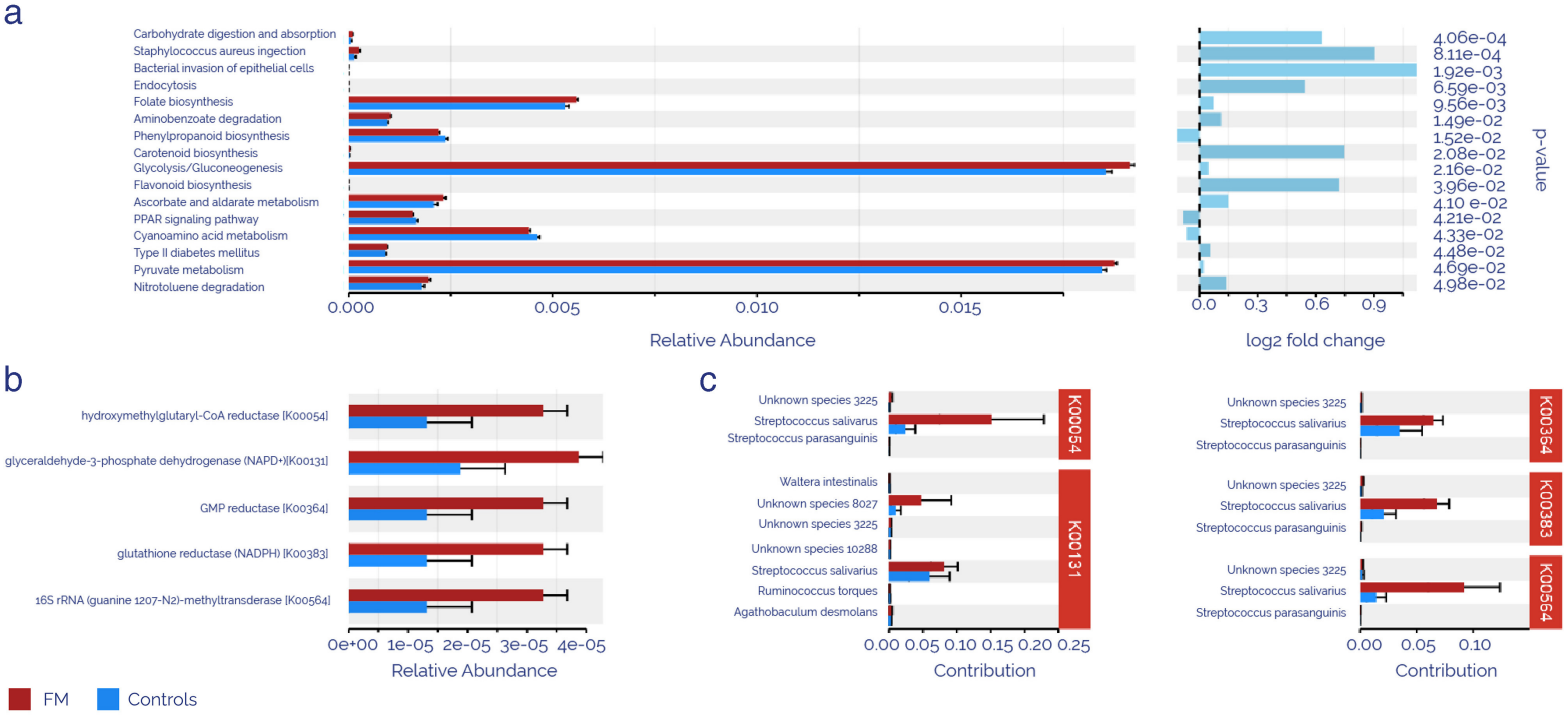
Predicted KEGG function analysis using PICRUSt. **(a)** Significantly enriched pathways according to the KEGGdatabase. **(b)** Top 5 significantly enriched enzyme activities. **(c)** zOTUS contributes to the enrichment of enzymatic activities. The red color represents the FM group, and the blue color represents the control group.

PICRUSt analysis was applied to zOTUS abundances using the KEGG database to determine whether the taxonomic differences between intestinal microbiota from FM and controls might cause functional changes. The results showed that 17 KEGG pathways were significantly different between patients and controls (Figure 3a). The FM microbiota exhibited increased carbohydrate, ascorbate, pyruvate metabolism, bacterial infections, endocytosis, and aminobenzoate degradation, as well as folate, carotenoid, and flavonoid biosynthesis, type II diabetes mellitus, and glycolysis/gluconeogenesis. Conversely, the FM microbiota suggested reduced pathways were phenylpropanoid biosynthesis, PPAR signaling pathway, and cyanamide acid metabolism (*p* < 0.05). Enzyme activity prediction (Figure 3b) highlighted the top 5 activities according to their *p-*values: hydroxymethylglutaryl-CoA reductase, glyceraldehyde-3-phosphate dehydrogenase (NADP+), GMP reductase, glutathione reductase (NADPH), and 16S rRNA (guanine1207-N2)-methyltransferase, all of which showed statistically significant upregulation in the FM group. An exhaustive analysis of zOTUS contributing to the enrichment of these enzymatic activities identified *Streptococcus salivarius* as the primary cause of this alteration, participating in all the enzymatic pathways identified.

### Multi-omics comparison

#### Correlation heatmaps

**Figure 4:**
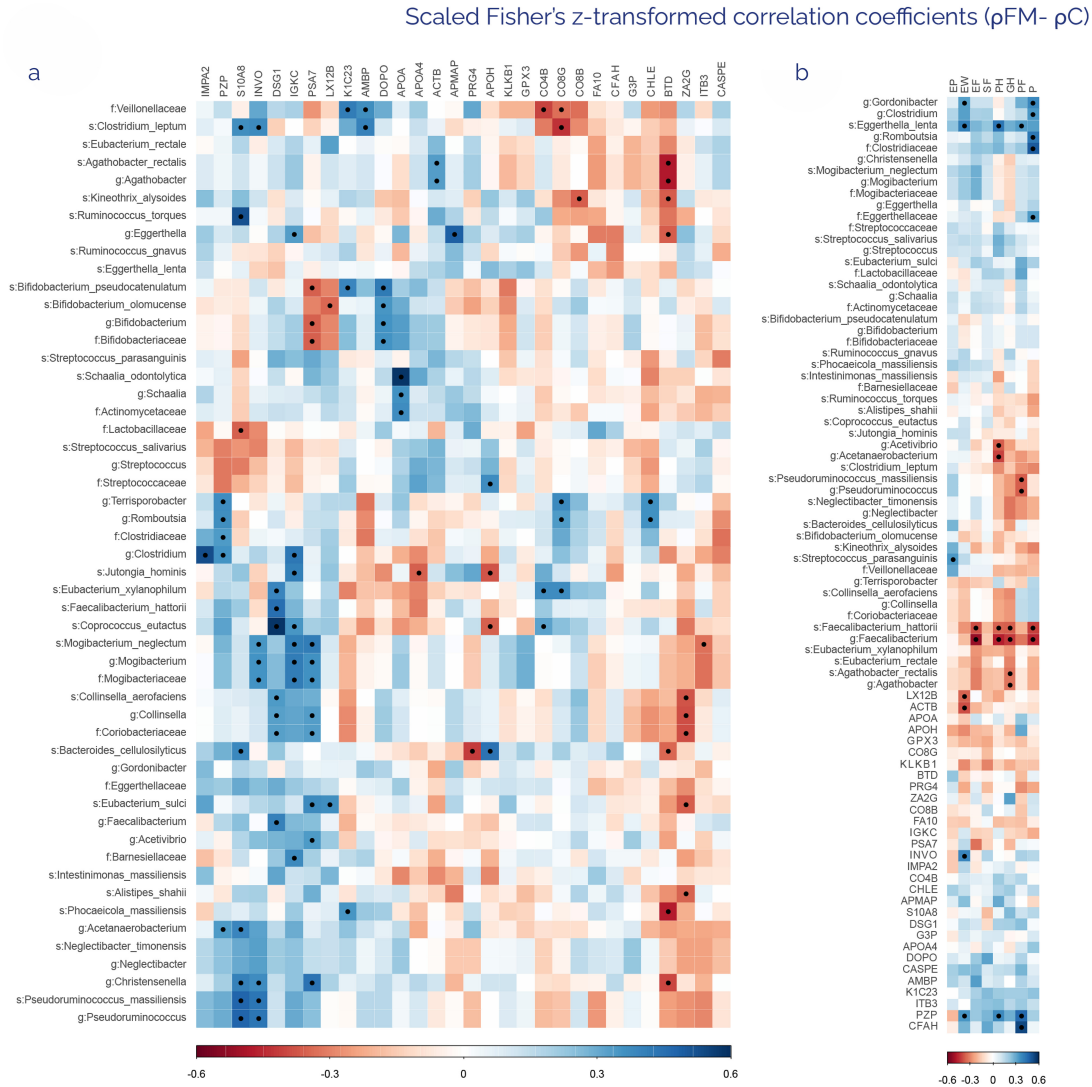
Heatmaps of scaled Fisher's z-transformed correlation coefficients (ρ) from the comparison of FM-related ρ vs. C-related ρ in different variables. The resulting and plotted ρ come from deducting FM-related ρ for a specific variable comparison to the C-related ρ for this same variable (ρFM- ρC). **(a)** Heatmap of Fisher's z-transformed ρ from ρFM-ρC between metagenomic zOTUs and proteomic markers with differentiated abundances. **(b)** Heatmap of Fisher's z-transformed ρ from ρFM-ρC between differentiated multi-omics results and SF36 clinical indices: emotional problems (EP), emotional wellbeing (EW), vitality-energy/fatigue (EF), social functioning (SF), physical health (PH), general health (GH), physical functioning (PF), and Pain (P). The heat map is sorted based on hierarchical clustering. Blue indicates positive correlations, whereas red indicates negative correlations (−0.6 < ρ > 0.6), with color saturation directly linked to ρ's absolute value. Those correlations with a statistically significant difference are marked by a black dot (Benjamini–Hochberg FDR < 0.05).

To explore the potential relationship between intestinal microbiota composition and the host plasma proteome profile, differences in ρ were studied between the FM and C group for each comparison. To depict the potential use of markers identified in both analyses as a diagnostic tool, zOTUs and proteins with statistically significant differences, independent of FC values, were correlated (Figure 4a). Several microbial taxa clustered due to their association with specific proteins. Interestingly, *Bifidobacterium pseudocatenulatum* and *Bifidobacterium alomucense* correlated with PSA7 (FM/C ratio: 1.37), LX12B (FM/C ratio: 1.33), and DOPO (FM/C ratio: 1.78), a solid result in phylogenetic terms, since the genus (*Bifidobacterium*) and the family (Bifidobacteriaceae) exhibited the same trend. This also occurred in other species like *Mogibacterium neglectum, Collinsella aerofaciens* or *Schaalia odontolytica* with their genera and families, respectively, consistently showing the relationship between them and the above-mentioned proteins. Moreover, certain proteins showed numerous correlations with taxonomic clusters. That was the case of PSA7 (FM/C ratio: 1.37), which had an inverse proportional correlation with zOTUs from the Bifidobacteriaceae family, and a positive one with zOTUs from the Coriobacteriaceae and Mogibacteriaceae families. So far, these two families have formed one of the most robust clusters due to their correlation with DSG1 (FM/C: ratio: 0.6), IGKC (FM/C ratio: 0.78), and PSA7 (FM/C ratio: 1.37) simultaneously. Another protein that correlated negatively with several zOTUs was BTD (FM/C ratio: 0.86), which showed similar negative correlations with the genera *Agathobacter, Eghertella*, and *Christensenella*, in addition to the species *Bacteroides cellulosilyticus* and *Phocaeiocola massiliensis*.

To determine which differences could be specifically associated with the quality of life of the participants, the correlations between the SF36 health questionnaire items and the omics data were represented (Figure 4b). Metagenomics showed the highest number of variable correlations with health scores, obtaining 15 zOTUs. A cluster composed mainly of zOTUs of the genus *Faecalibacterium* correlated negatively with most questionnaire items, including vitality, physical health, general health, and pain. In this sense, pain was the variable that showed a higher number of positive and negative correlations with proposed fecal markers. Although proteins had only 5 correlations, most of them were linked to the mental health score, a crucial factor in FM disorder.

Regarding the FIQR questionnaire, ρ for the omics results of FM patients and FIQR scores was calculated. The results were not significant for any of the variables, although a weak correlation (|ρ| > 0.2) suggests that depression was the item that correlated with a greater number of zOTUs, with a total of 9. Furthermore, pain showed a direct proportional correlation with all zOTUs of the Mogibacteriaceae family. As for proteins, only CASPE (FM/C ratio: 1.24) correlated negatively with stiffness, which correlates positively with the Streptococcaceae family at the same time (data not shown).

#### ROC curves

**Figure 5:**
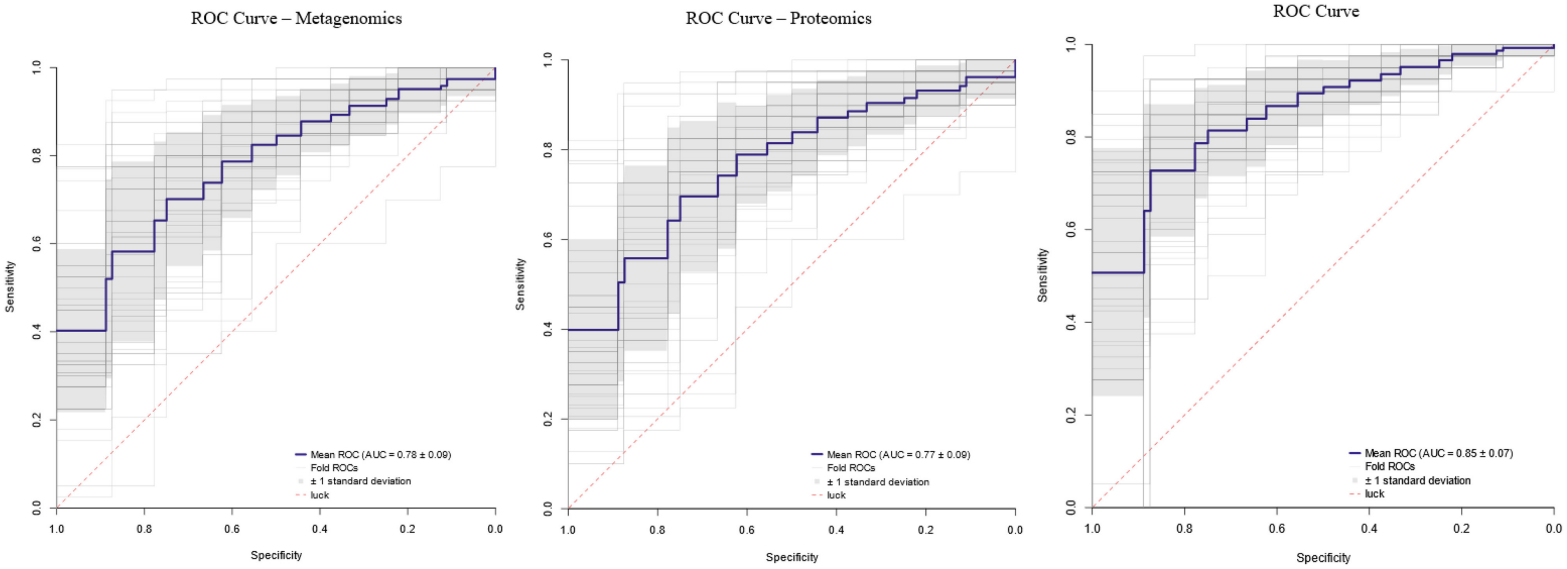
ROC curves. Area under the curve (AUC) values for each omics dataset and their combination.

The diagnostic performance of the metagenomic and proteomic datasets was evaluated using supervised machine learning on 242 samples (199 FM patients and 43 controls). Models trained on each omics layer separately achieved good classification performance, indicating that both microbial and proteomic profiles contain relevant, non-redundant information for distinguishing FM patients from controls. However, the best results were obtained when both datasets were integrated into a single model, which achieved a mean AUC of 0.85 ± 0.07 across repeated stratified cross-validation (Figure 5). This integrative model, which leveraged all statistically significant microbial taxa and proteins, consistently outperformed the single-omic approaches. These findings highlight the complementary nature of the two omics layers and support the use of a combined multi-omics panel as the most effective strategy for discriminating FM patients from controls.

## Discussion

FM is a chronic pain-related disorder that is still underdiagnosed due to the lack of molecular markers. The present study is focused on the search for these markers in the plasma proteome and intestinal microbiota. The results showed a significant differential pattern of 30 proteins and 19 bacterial taxa between FM patients and controls, and a significantly higher α diversity in controls compared to patients, suggesting a worse intestinal environment and, consequently, a weaker intestinal and general health in FM.

Main symptoms of FM (pain and cognitive impairment) suggest an evident and deleterious nervous system disruption. In fact, several authors have agreed on classifying this disorder as one of the most common conditions among the so-called central sensitization syndromes (Boomershine, 2015), which are characterized by a CNS imbalance that affects the homeostasis of the whole nervous system. This altered neurological performance has been demonstrated in a substantial number of studies utilizing various imaging, electrodermal, and molecular techniques. For instance, different groups have found that nerve fibers participating in nociception in FM patients are less abundant, thinner, and even abnormally hyperexcited when compared with controls (Serra et al., 2014; Vecchio et al., 2020). Similarly, autonomic nervous system-dependent processes such as sweating have been reported as compromised by pathological traits in nerve fibers identified through electrodermal measurements (Reyes del Paso and de la Coba, 2020; Falco et al., 2024), adding further evidence to support the hypothesis of a complete nervous system failure in these patients.

At the molecular level, the long list of neurological imbalances is usually accompanied by chaotic neuroimmunological signaling. An extensive and varied record of altered molecular signaling in the immune system has been reported (Ovejero et al., 2020; Verma et al., 2022; Maes et al., 2024; Goebel et al., 2021), resulting in marked neuroinflammation, a common finding in FM patients (Littlejohn and Guymer, 2018; Mueller et al., 2023). In line with this hypothesis, nervous system impairment was also inferred by proteomic analysis in our cohort, with CHLE (FM/C ratio: 0.85), DOPO (FM/C ratio: 0.85) and G3P (FM/C ratio: 0.82) as affected proteins among others (Table 3); the first and the second, participate actively in the regulation of cholinergic and dopaminergic synapses (Darvesh et al., 2003; Silman, 2021; Rush and Geffen, 1980), and the third one, is annotated in the GO term neuron apoptosis process (GO:0051402). In fact, the role of G3P protein (GAPDH) seems to be essential in the gut-brain-mitochondrial axis, as discussed in the upcoming paragraphs. Inflammation traits were as well identified in our study, since some proteins among the 30 differentially expressed in the FM patients were narrowly related with pro-inflammatory pathways (Figure 1b), such as acute phase signaling, with AMBP (FM/C ratio: 1.10), APOH (FM/C ratio: 1.32), CO4B (FM/C ratio: 0.86), and KLKB1 (FM/C ratio: 0.88) as altered proteins; and IL-12 signaling and production in macrophages, represented by APOA4 (FM/C ratio: 0.83), IGKC, (FM/C ratio: 0.78), APOA (FM/C ratio: 0.83), and S100A8 (FM/C ratio: 1.43) as Table 3 showed. The last one, a component of calprotectin, has recently been proposed as a biomarker for rheumatoid arthritis and FM (Inciarte-Mundo et al., 2022; Vega-Ramírez et al., 2024), given its implication in the activation of innate immunity and inflammation.

In FM patients, the presence of neuroinflammation has been proposed as a multifactorial process driven by the co-occurrence of low vitamin D levels, mitochondrial dysfunction, and intestinal microbiota dysbiosis (Vasquez, 2016). In this sense, our proteomic results identified four proteins participating in the 24-dehydrocholesterol reductase (DHCR24) signaling pathway, according to IPA: AMBP (FM/C ratio: 1.10), APOA4 (FM/C ratio: 1.25), APOH (FM/C ratio: 1.32), and CO4B (FM/C ratio: 0.86). DHCR24 is the final enzyme in the biosynthesis pathway of cholesterol, a ubiquitous molecule that serves as a precursor to several by-products participating in a wide variety of physiological and pathological pathways, such as the metabolism of lipids and vitamin D, the synthesis of steroid hormones, and liver X-receptor (LXR)/retinoid X-receptor (RXR) activation, among others (Warren et al., 2021). LXR/RXR activation appears to be supported by stronger evidence in previous studies from our group and other authors. In the present study, the LXR/RXR activation pathway is the most differential canonical pathway between FM and C groups according to the *p-*value (Figure 1a), and is represented by the differential expression of AMBP (FM/C ratio: 1.10), APOA4 (FM/C ratio: 1.25), APOH (FM/C ratio: 1.32), CO4B (FM/C ratio: 0.86), APOA (FM/C ratio: 0.83), and S100A8 (FM/C ratio: 1.43), with a clear tendency toward upregulation. LXR/RXR receptors are steroid- and lipidic metabolites-dependent transcription factors involved in biological processes as diverse as lipid metabolism, immune system maintenance, neurobiological homeostasis or reproductive health homeostasis. Not in vain, this route has been significantly enriched and activated in numerous cohorts of FM patients in previous research (Ramírez-Tejero et al., 2018; Han et al., 2020; Frigo et al., 2021; Mohapatra et al., 2024), leading to altered lipid metabolism. Since steroid synthesis takes place inside the mitochondria, starting from cholesterol, the complete disruption found in lipid and steroid metabolism of FM patients that, at the end, alters the performance of the neuroendocrine system might be mainly triggered by this organelle.

Not surprisingly, mitochondria may be the common link among the various symptoms experienced by FM patients. By far, the nervous and musculoskeletal systems have the highest energy demand in the body, and they have been found to be dramatically affected by mitochondrial dysfunction (Cantó-Santos et al., 2020). Similarly, mitochondria have been repeatedly mentioned as a dysfunctional organelle in FM, not only by the scientific community, but also in previous studies from our research group (Cordero et al., 2013; Martínez-Lara et al., 2020; Castro-Marrero et al., 2013; Sánchez-Domínguez et al., 2015; Gerdle et al., 2020; Alcocer-Gómez et al., 2015; Cordero et al., 2010, 2016), which makes perfect sense, considering that pain, fatigue, joint stiffness, muscle cramps, and neurocognitive disorders stand out as main features of FM. Specifically, our previous findings suggested a profound imbalance of the mitochondrial membrane-associated porin voltage-dependent anion channel 1 (VDAC1) in FM patients (Martínez-Lara et al., 2020; Ramírez-Tejero et al., 2023), which directly cooperates with G3P in a well-defined classical mitochondrial-related apoptosis pathway (Tarze et al., 2007). Additionally, VDAC1 was identified in the plasma proteome analysis of our cohort, although it did not show statistically significant differences between groups (data not shown). This result may be due to technical limitations of the nLC-MS/MS approach, as even though a depletion step was introduced, the most abundant proteins could mask subtle changes in the less present ones. However, G3P was differentially expressed in FM patients, as shown in Table 3. This specific enzymatic activity appeared as the top functionally decreased activity in the intestinal microbiome of FM patients (Figure 3b) and is involved in the main pathways altered in FM patients, as indicated by our data. For this reason, the G3P enzyme and its by-products emerge as a promising diagnostic and therapeutic target for FM patients, and its enzymatic increase inferred from the metagenomic functional analysis could be a counteracting response to the lower plasma levels in FM patients.

Sticking to the mitochondrial environment, several other molecular functions take place inside this organelle, apart from energy metabolism. Specifically, mitochondria have been linked to oxidative stress and subsequent inflammatory processes in a wide range of pathological conditions (Chen et al., 2023). Among the differentially expressed proteins in FM patients participating in this study, some proteins related to these processes were found. For instance, GPX3 (FM/C ratio: 0.85), LX12B (FM/C ratio: 1.33), and APOA4 (FM/C ratio: 1.25) differences shown in Table 3, suggested a deficiency in oxidative stress defense as well as an increased lipid peroxidation, results already reported in FM and even linked to pain intensity and inflammation in those patients (Assavarittirong et al., 2022; Meeus et al., 2013; Akkuş et al., 2009; Ozgocmen et al., 2006). In line with these previous findings, some of the top*-*rated canonical pathways according to the *p-*value were complement cascade and complement system, implying proteins such as CO4B (FM/C ratio: 0.86), CO8B (FM/C ratio: 0.89), CO8G (FM/C ratio: 0.81), CFAH (FM/C ratio: 1.15), and IGKC (FM/C ratio: 0.78). This molecular pathway is narrowly related to chronic inflammation (Rawish et al., 2021).

All the previously identified imbalances found in our study could have a common link in the intestinal microbiome, defined as the unique signature of microbes, their genes, and metabolites, as well as their interaction with the host. Intestinal dysbiosis has been identified as a main source of CNS-related syndromes and symptoms, often via the vagus nerve and immune system (Rutsch et al., 2020). This connection has already been evaluated in FM, finding a low vagal nerve tone that might be related to pain and inflammation, as reported also for other conditions (Martins et al., 2021). One of the latest hypotheses links FM with this exact connection, dysbiosis being the main triggering cause of CNS symptoms. In FM patients, those deviations have lately been blamed for associated complications, such as irritable bowel syndrome (Garofalo et al., 2023) or psychological distress (Nhu et al., 2024). Besides that, a fast-growing hypothesis for some FM-overlapping sex-related conditions, such as endometriosis, breast and endometrial cancer, points to the role of β-glucuronidase activity from intestinal microbiota metagenome (estrobolome), specifically from the phyla Bacteroidetes (Bacteroidota) and Firmicutes (Bacillota) (Pollet et al., 2017), as the primary source of estrogens disruptions (Kwa et al., 2016; Baker et al., 2017; Ervin et al., 2019; Komorowski and Pezo, 2020; Salliss et al., 2022; Siddiqui et al., 2022; Sobstyl et al., 2022; Pai et al., 2023; Hu et al., 2023). This hypothesis reinforces the crucial role of the intestinal microbiota in a considerable list of human diseases and syndromes. In terms of hormone signaling, and following the estrobolome hypothesis, cortisol, estradiol, and progesterone levels have been investigated in FM patients and *in vitro* models; however, the findings on the impact of these hormones on pain and neuropsychological complications were preliminary and contradictory (Okifuji and Turk, 2006; Rus et al., 2016; Schertzinger et al., 2018; Hernandez-Leon et al., 2018; Lin et al., 2021).

Our results revealed a differential pattern in intestinal microbiota composition in FM patients, as shown in the volcano plot resulting from the metagenomic analysis (Figure 2c), with a total of 19 bacterial taxa that were differentially expressed. Specifically, at the phylum level, the distribution of zOTUS is as follows: 12 *Bacillota*, six Actinobacteria, and one Bacteroidota. *Streptococcus salivarius* appeared as the most differential bacterium between the FM and C group, with the lowest *p-*value and the highest FC. Interestingly, this bacterium has demonstrated regulatory properties for immunological and inflammatory responses *in vitro* (Kaci et al., 2014), suggesting that this marked difference may represent an outstanding research hypothesis for further studies. Several other species, such as *Mogibacterium neglectum, Streptococcus parasanguinis, Schaalia odontolytica, Pseudoruminococcus massiliensis, Ruminococcus gnavus*, and *Bifidobacterium pseudocatenulatum* showed the same trend as *S. salivarius*. On the other hand, *Bacteroides cellulositycus* and *Intestinimonas massiliensis* were found to be less abundant in FM patients, according to our results. To our knowledge, this is the first paper to report differential abundance in FM patients for bacteria from these genera, except for *Bifidobacterium* and *Streptococcus*. The first one was previously identified as a reduced genus in FM patients (Clos-Garcia et al., 2019), and the second one showed differences between men and women with FM in a pilot, descriptive study of our group (Ramírez-Tejero et al., 2023). In this regard, different research groups have demonstrated differentially abundant bacterial groups in the gut of FM patients compared with controls. (Minerbi et al. 2019) reported a distinct microbiota signature in FM patients, composed of a high prevalence of bacteria from the Firmicutes phylum. Besides, species such as *Prevotella copri, Faecalibacterium Praustnitzii*, and *Bacteroides uniformis*, classically associated with a healthy gut, showed less abundance in those patients. In a parallel study, (Clos-Garcia et al. 2019) found a similar pattern, where beneficial bacteria, such as *Bifidobacterium* spp. and *Bacteroides* spp., were clearly less abundant in patients compared to controls, as mentioned before. Moreover, this group demonstrated some correlation between these differences and the concentration of several metabolites with a crucial role in CNS signaling. Our results showed a striking coincidence, since *Bacteroides cellulosyticus* appeared with the same exact tendency as that found in both studies for their genus relatives. Several species from this genus have exerted a remarkable ability to participate in γ-aminobutyric acid (GABA) synthesis in the human gut (Otaru et al., 2021). GABA is the primary calming CNS neurotransmitter, and previous authors have demonstrated that it is present in the brain at a lower concentration in FM patients compared to controls, while its precursor and main excitatory neurotransmitter, L-glutamate, exhibits the opposite behavior (Minerbi et al., 2019; Otaru et al., 2021). In this sense, the generalized *Bacteroides* spp. deficiency that seems to be present in FM patients might be a promising therapeutic target for CNS imbalances in them, although further research is needed.

Functional prediction with PICRUSt of bacterial differences in our FM cohort provided promising results. Several pathways with a marked link to mitochondria were enriched in those patients. As shown in Figure 3a, glycolysis/gluconeogenesis and pyruvate metabolism were, by far, the most affected routes. Additionally, some vital routes for mitochondrial health were also compromised. For instance, the PPAR signaling pathway and folate biosynthesis (Xiu and Field, 2020; Mello et al., 2016). In line with this hypothesis, typically mitochondrial enzymatic activities also showed a differential behavior in ontology prediction based on intestinal dysbiosis found in these patients. That was the case of hydroxymethylglutaryl-CoA reductase (K00054) and glyceraldehyde-3-phosphate dehydrogenase (NADP+) (K00131). Mitochondrial dysfunction could be a triggering factor, according to further PICRUSt predictions, since antioxidant activities, such as glutathione reductase (NADPH) (K00383), also appear to be compromised. Taking into account that plasma results were suggesting a mitochondrial blockade, microbiota might be reacting as a counterbalance to this deficiency.

The multi-omics approach provided valuable insights into the relationships between proteins, bacteria, and health questionnaire results. In this sense, as shown in Figure 4, Fisher's z-transformed ρ highlighted evident differences in protein-to-bacteria and protein/bacteria-to-health questionnaire scores between controls and patients. Thus, strong positive differences in correlations were found between DSG1 and IGKC proteins and several bacterial genera, including *Clostridium, Eubacterium, Faecalibacterium, Coprococcus, Collinsella*, and *Mogibacterium*, all of which belong to the phylum Firmicutes. Desmogleins (DSG1 and DSG2) are essential proteins in cell-to-cell adhesion, serving as the primary components of desmosomes in tissues subjected to strong mechanical stress, such as the intestinal barrier layer (Gross et al., 2018; Sherrill et al., 2014). IGKC, as the constant region of the light chains from all immunoglobulin types, participates in a wide range of adaptive immune responses. Combined, these findings suggest that, in FM patients, the highest abundance of these genera may trigger an intestinal barrier breakdown, followed by an immunological response, whereas controls do not experience such a disturbance. Since FM has been classically linked to several gastrointestinal challenges (Slim et al., 2015), the intestinal microbiome composition of these patients should be a clinical indicator in their initial evaluations, as well as a therapeutic target to address. Furthermore, DOPO protein exerted a marked positive correlation with the Bifidobacteriaceae family, specifically with *Bifidobacterium pseoudocatenulatum* and *Bifidobacterium alomucense*. According to our results, the increase in these bacteria is linked to a higher abundance of Dopamine beta-hydroxylase, which is responsible for converting dopamine (a pleasure neurotransmitter) to noradrenaline (a concentration neurotransmitter). The link between these bacteria and dopaminergic signaling has been widely depicted in the scientific literature (Hamamah et al., 2022). For instance, in murine models, the supplementation with *Bifidobacterium infantis* elevated the levels of noradrenaline after maternal separation (Desbonnet et al., 2010). Whether these differential effects of *Bifidobacterium* spp. on dopamine/noradrenaline signaling could be acting as a gut-brain axis regulator checkpoint in FM symptoms needs to be addressed in further studies. An additional relevant finding concerns *Bacteroides cellulosyliticus* and the marked and opposite relationship established with the proteins PRG4 and APOH, both of which have higher levels in FM patients (Table 3). According to the heatmap of correlation differences, the presence of *B. cellulosyliticus* is somewhat related to PRG4. This proteoglycan plays a significant role in joint lubrication, but is also implicated in the management of neuroinflammation by interfering with TLR2/4 signaling (Bennett et al., 2021). In parallel, this bacterium showed a positive difference in correlations with APOH, which was previously described as a biomarker for antiphospholipid syndrome (Sibilia, 2003), a condition that worsens FM symptoms when it appears as a comorbidity (Costa et al., 2011). Given that, as previously mentioned*, Bacteroides* spp. was found to be a reduced genus in FM patients in both previous studies and our results, its differential correlation with these two proteins could be proposed as a molecular mechanism affected in patients.

Finally, regarding health questionnaires, the highest differences between FM and C correlation coefficients are shown by CFAH and *Faecalibacterium hattori*. The former showed positive differences with physical function, while the latter showed negative differences with energy/fatigue, physical health, general health, and pain, as shown in Table 2. Hence, CFAH and *Faecalibacterium hattori* could be potential therapeutic targets to address, thereby enhancing patients' quality of life.

## Conclusions

This is the first multi-omics approach for FM patients, highlighting the feasible role of the gut-brain-mitochondrial axis in FM etiopathogenesis in such a large cohort. This study identifies several promising markers for classifying patients with FM. Plasma and fecal multi-omics analysis and its integration into an algorithm allows discrimination between cases and controls with high AUC values, pointing to an intricate and multifactorial connection between gut microbiota and mitochondria-derived oxidative stress and inflammation, with glyceraldehyde-3-phosphate dehydrogenase and *Streptococcus salivarius* as leading actors. All biomarkers identified in this study could play an important role in the disease, suggesting their potential as prognostic, diagnostic, and therapeutic targets for personalized, preventive, or palliative/curative treatment of FM in the future. However, further blinded studies linking these potential biomarkers to FM could define a specific signature of women suffering from FM, which would be very useful for developing a robust diagnostic tool and focusing on probiotic-based treatments or other effective procedures for the patient.

## Data Availability

The datasets presented in this study are publicly available. The metagenomic data are deposited in GSA-Human under the accession number HRA009721 (BioProject accession: PRJCA032811), available at https://bigd.big.ac.cn/gsa-human/browse/HRA009721. The proteomic data are deposited in the PRIDE database under the ProteomeXchange accession number PXD059894, with the project webpage available at https://www.ebi.ac.uk/pride/archive/projects/PXD059894 and the FTP download at https://ftp.pride.ebi.ac.uk/pride/data/archive/2025/09/PXD059894.
