# Supplementary material for: Fibromyalgia diagnosis from a multi-omics approach: a gut feeling
Source: Front Microbiol. 2025 Oct 2;16:1641185. doi: 10.3389/fmicb.2025.1641185 (PMC12528038; doi:10.3389/fmicb.2025.1641185)
Supplement: Supplementary file 1 [file Table_1.pdf]

Table S1. Technical validation of biomarkers discovery and functional analysis of antioxidant capacity. Proteins levels were quantified by Enzyme-Linked Immunosorbent Assay (ELISAS), microorganisms' abundances were assessed via TaqMan quantitative Polymerase Chain Reaction (qPCR) and total antioxidant capacity was determined through electrochemistry assay. Mean values are shown for the control (C) and fibromyalgia (FM) groups. Fold Change represents the FM/C ratio. Two statistical tests were applied: Student's *t*-test and Mann–Whitney *U* test.

**Biomarker validated**	**Technique**	**Mean C**	**Mean FM**	**Fold Change (FM/C)**	**p-value (*t*-test)**	**p-value (Mann Whitney *U*)**
**Beta-2-glycoprotein 1 (APOH)**	ELISA (ng/ml)	1262.01 (*n* = 7)	1560.15 (*n* = 25)	1.24	0.42	0.43
**Apolipoprotein A-IV (APOA4)**	ELISA (ng/ml)	54073.78 (*n* = 9)	73753.09 (*n* = 23)	1.36	< 0.005	< 0.005
Streptococcus parasanguinis	qPCR (relative Ct)	5.62 (*n* = 43)	4.44 (*n* = 51)	0.79	< 0.005	< 0.005
Streptococcus salivarius	qPCR (relative Ct)	4.01 (*n* = 43)	3.00 (*n* = 51)	0.75	< 0.00001	< 0.00005
**Total Antioxidant Capacity**	Colorimetri c FRAP	11.40 (*n* = 43)	10.90 (*n* = 199)	0.95	0.91	0.84
